# Newborn Screening for Critical Congenital Heart Disease in a Low-Resource Setting; Research Protocol and Preliminary Results of the Tanzania Pulse Oximetry Study

**DOI:** 10.5334/gh.1110

**Published:** 2022-05-26

**Authors:** Naizihijwa Majani, Pilly Chillo, Martijn G. Slieker, Godwin Sharau, Vivienne Mlawi, Stella Mongella, Deogratias Nkya, Sulende Khuboja, Gideon Kwesigabo, Appolinary Kamuhabwa, Mohamed Janabi, Diederik Grobbee

**Affiliations:** 1 Jakaya Kikwete Cardiac Institute, Dar es Salaam, Tanzania; 2 Julius Global Health, the Julius Center for Health Sciences and Primary Care, University Medical Center Utrecht, the Netherlands; 3 Muhimbili University of Health and Allied Sciences, Dar es Salaam, Tanzania; 4 Department of Paediatric Cardiology, University Medical Center Utrecht, the Netherlands

**Keywords:** critical congenital heart disease, pulse oximetry, newborn screening, low-resource setting

## Abstract

**Background:**

Critical Congenital Heart Disease (CCHD) is the leading cause of early new-born mortality. Its early detection and intervention is crucial for the survival of affected new-born. Pulse Oximetry (POX) has shown to be one of the feasible, accurate and cost-effective tools in screening CCHD in developed nations, it is yet to be practiced and established as standard of care in a low-resource setting.

**Objectives:**

This paper reports on the research protocol and preliminary results of an ongoing study regarding the performance of POX in detecting CCHD in new-borns in a low resource setting. Secondary objectives include investigating the burdens of CCHD and outcome at 12 months of age.

**Methods:**

The Tanzanian Pulse Oximetry Study (TPOXS) is a prospective cohort study which plans to enrol 30,000 mothers and new-borns delivered at two referral hospitals in Tanzania. New-borns are offered POX test 12 hours after birth, those positively undergoes echocardiography examinations. Confirmed with CCHD are placed under observation for up to first birthday.

**Results:**

During a 5-months pilot period, a total of 1,592 infants at the Muhimbili National Hospital, received POX test .65% of them were post-caesarean section and 52% being male. Most babies delivered through Spontaneous Vertex Delivery (SVD) were promptly discharge and did not get screened. The detection-rate of CCHD was 2.5 per 1,000 live births (at 95% confidence interval [CI] 0.9 to 6.7 per 1000 live birth); with a POX false positive rate of 0.6%. Seven false-positive infants out of 10 were found to carry significant other neonatal conditions, including persistent pulmonary hypertension of the new-born, transient tachypnoeic and neonatal sepsis.

**Conclusion:**

This paper provides the protocol of the ongoing TPOXS with the preliminary results showing prevalence matching closely the global data. It shows acceptability of POX screening for CCHD in a well-prepared low resource setting.

**Highlight:**

This study addresses the utilization of pulse oximeter in detecting critical congenital heart disease (CCHD) in a low-resource setting (such as sub-Saharan African countries).

## Introduction

Congenital heart disease (CHD), a structural abnormality of the heart and great vessels, is a global burden with nearly 12 million people living with [1,2]. About a quarter of births with CHD are classified as critical congenital heart disease (CCHD) requiring immediate intervention preferably within initial year of birth to ensure survival [3]. Estimates in Europe and USA place an overall CCHD incidence at 2–3 per 1,000 live births [4,5]. High fertility rates in sub-Saharan Africa are tied to respective high CHD prevalence [1]. However, low documentation of birth prevalence of both CHD and CCHD is reported. In Tanzania, for example, nearly 2 million total annual live births carry a substantial burden of CHD and as extrapolated from world prevalence data, an estimated 13,600 children are born annually with CHD, with about 4,000 of them probably having CCHD.

POX screening has been confirmed as a promising screening modality for CCHD by offering timely identification of infants with CCHD and thereby minimizing morbidity and mortality [11]. Consequently, screening for CCHD is considered standard of care across the developed world, with the Nordic region, United States, Switzerland, and United Arab Emirates approaching universal screening [12]. The developing nations of Africa, South and Central America exhibit different stages of organizing universal CCHD screening [11,12]. In sub-Saharan Africa, there are no current ongoing country-wide universal screening programmes for CCHD.

In a low-resource setting, POX adoption is debatable. Adequate evidence doesn’t exist yet to support POX feasibility and the capacity of health systems to handle an additional screening task to add on to the already burdened health-care services [13,14,15]. These reasons highlight on the criticality of this debate for sub-Saharan Africa less developed health systems. However, the ongoing economic progress in many developing countries translates into emergence and availability of cardiac centres due to the presence and expertise of health-care workers, health-care funding and equipment. It is, therefore, essential to look at the performance of screening programs in the region, its impact on neonatal health and to answer questions regarding the utility and need for such POX screening programs [19].

The TPOXS is designed to assess the performance of newborn POX screening in a larger sample of >30,000 live births in a typical low-resource setting. Furthermore, the study aims to determine the birth prevalence of CCHD, study risk factors for CCHD, and document the outcome of one-year old children with CCHD.

## Methods

### Study Setting

This study is conducted in Dar es Salaam City located 24 m above sea level by the Indian Ocean. The two study sites represent tertiary and secondary care government-owned hospitals.

The Muhimbili National Hospital (MNH) is a tertiary referral centre for referred patients from and outside Dar es Salaam. Approximately 6,000–10,000 annual deliveries occur at MNH. Women especially those with uncomplicated delivery are discharged within 24 hours. It has an Intensive Care Unit (NICU), a kangaroo mother care, and a neonatal ward for 100 neonates.

Mwananyamala Refferal Hospital (MRH) is a regional and secondary care facility located about 6 km away from MNH. There are approximately 7,000–10,000 annual deliveries. On average, similarly, women are discharged within 24 hours of delivery. MRH is randomly chosen from three other Dar es salaam regional hospitals. It has a 10-bed NICU, a kangaroo mother care, and a neonatal ward for 30 neonates.

### Study Design, sample size and duration

This two-year (October 2020 to September 2022) prospective cohort study is planned to screen a total of 30,000 childbirths for the detection of risk factors of having CCHD (with higher odds 2.0 or more) from other maternal factors as advanced age, chronic gestational, infectious and non–infectious diseases, fever, non-use of folic acid, alcohol abuse and obesity, at the power of 80%.

### Inclusion and exclusion criteria

During the study period, all full-term or near term (GA > 35 weeks) newborns delivered at the two hospitals and admitted in regular newborn nurseries >12 hrs of age are eligible for study enrolment. All babies delivered before the 35 weeks gestational age, those older than seven days, those sick and admitted at NICU immediately after birth due to severe illness or severe malformations will be excluded. This is due to premature newborns undergoing multiple investigations during admissions and being less likely to be missed for CCHD evaluation. However, throughout the study period, the study team will be visiting the premature ward to ascertain any CCHD diagnosis before due hospital discharge.

### Study Site Preparation

Prior to initiation of screening, sites are visited and checked for readiness, including staff and screening tools availability (Appendix 1 has a list of screening tools). A two-hour screening protocol training is conducted for antenatal wards nurses. Afterwards, a test questionnaire for competence is administered requiring a score of above 95%. A list of eligible nurses is maintained both at the station and with the study team. Nurses who have failed the test criteria receive feedback and are encouraged to attempt the test. A pilot study is then conducted at the site to validate the questionnaire. Designated study team members visit screening sites daily to offer support, ensure protocol adherence, identify training needs, and collect filled questionnaires for processing.

### Data Collection Procedures

#### Enrolment into the study

All eligible children are enrolled into the study by a shift nurse 12 hours after delivery, immediately before the discharge and/or as soon as the mother has signed the informed consent.

#### Pulse Oximetry Screening

**Figure 1:**
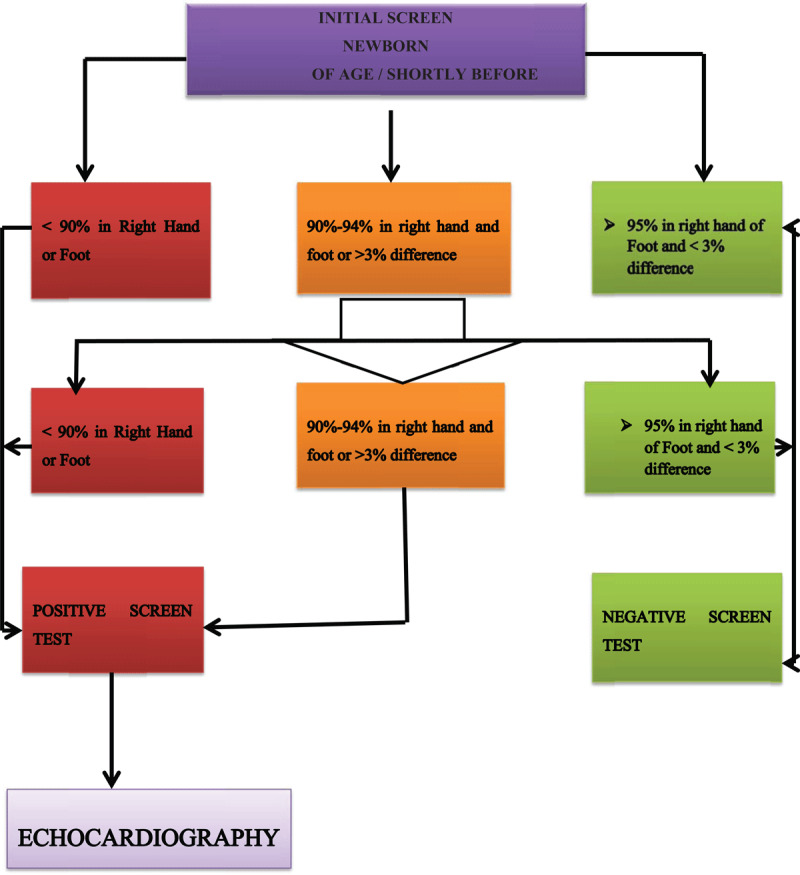
Modified Algorithm Newborn Screening CCHD. Adopted from Mahle, the USA screening program [2,9,10].

To all newborns, POX is taken first from the right hand plus either foot with the results entered into the predefined data collection form. POX is done using a handheld Bistos 4.3” device (as depicted in Attachment 2) specifically designed for children and newborns for accurate POX performance during motion and low perfusion. A well-fitted neonatal sensor used at all times is secured by a tape to the neonate’s finger, foot or palm. After exactly a minute (to ensure a stabilized reading for 5 seconds) a record is taken from the highest reading flashing on the device. All babies screened have a sticker on their postnatal card for immunization with a note to the investigator in the event of subsequent diagnosis of CCHD. The algorithm of the POX screening is provided in ***Figure 1***.

#### Confirmation of CCHD

All positive POX screen results get a detailed echocardiogram – using Vivid E95 GE echocardiography machine with a large 22” high-resolution OLED monitor integrated with C-sound and the M5Sc XD clear neonatal (12s 4.0–12.0 Mhz) using infant (6s 2.4–8.0 Mhz) transducers – to identify the presence of CCHD. The study site’s Principal Investigator (paediatric cardiologist) and assistant echocardiographer/paediatrician perform Echocardiography, which follows standard and familiar procedures of acquiring Echo-cardiograph images in newborns. The six Echocardiographers initially received a refresher course. Echo-cardiograph images are stored and sent for assessment to Utrecht University. Results are entered into a predefined data collection form to systematically collect information on cardiac anatomy.

#### Follow up

Follow up of newborns with CCHD is done at Jakaya Kikwete Cardiac Institute (JKCI) which is in very close proximity to MNH. It is government-owned and Tanzania’s top-most referral hospital for cardiac health. It has 150-bed capacity with all services, including percutaneous cardiac interventions for children and adults’ open-heart surgery. Since 2015, specialized paediatric cardiac surgeries are conducted at the Centre with total 810 open-heart surgeries for children conducted since inception. Paediatric intervention has grown from 70 to 200 cases between 2015 and 2019. Protocol for children with CCHD at the Institute dictates immediate stabilization with Prostin and Rashkind procedure, followed by fast-tracking to operating theatre.

Newborn CCHD cases receive 12 months observation, specifically at the age of six weeks, 24 weeks, and 12 months. The follow up whose findings are recorded in a predefined tool covers clinical status detailing physical examination together with inquiries about carried out procedures done and outcome.

Positive POX screen cases without CCHD on Echocardiogram are referred to the attending paediatrician and managed per established hospital protocol on hypoxia in children. Their final diagnosis is recorded and communicated to the investigating team. Information obtained is entered into a predefined data collection form. Negative POX tests receive at the age of six months telephone calls to ascertain a possible later CCHD diagnosis. The information is recorded in a predefined data collection form.

To account for any missed or false-negative POX results, physicians in the Department of Paediatric Cardiology at JKCI have been requested to notify study investigators of any new CCHD patient they attended to during the study period (i.e., from October 2020). These are being checked to determine origin from the two study hospitals.

#### Data handling and plan for analysis

Filled questionnaires are cross-checked for quality control. The collected data is coded, processed, and cleaned up for inconsistencies and outliers and then entered into Redcap (Research Electronic Data Capture) version 16 registered account for this study and later uploaded to be analyzed using STATA Statistical Package. Continuous variables are tested for normality distribution using normal probability plots and reported as mean ± SD or median and Interquartile range (IQR). The percentage is used for discrete variables. Matched groups will be compared using paired t-test, and unmatched groups will be compared using a two-sample student’s t-test. Categorical variables will be compared using a Chi-square test. Kaplan–Meir analysis will be used to ascertain survival probabilities at the end of one year of follow up. Cox proportional hazards regression modelling will be used to assess risk factors in the composite outcome of survival free from cardiac death and identify the factors independently associated with the composite endpoint. To determine whether predictors of all-cause mortality differ for various forms of CCHD, each covariate will be tested for interaction. P-value < 0.05 will be considered statistically significant. The odds ratio for the risk factor will be calculated. Sensitivity, Specificity, Positive Predictive Value (PPV) and Negative likelihood ratio will be calculated for the POX screening test.

#### Ethical Considerations

Ethical approval has been granted from the Muhimbili University of Health and Allied Sciences (MUHAS). Mothers/guardian receive clear communication of the study before enrolment to assure them that they can opt out of the study without consequence. Whenever mothers decline a written consent, the child is not enrolled and follows other routine postnatal care as per hospital protocol.

## Preliminary Results

**Figure 2:**
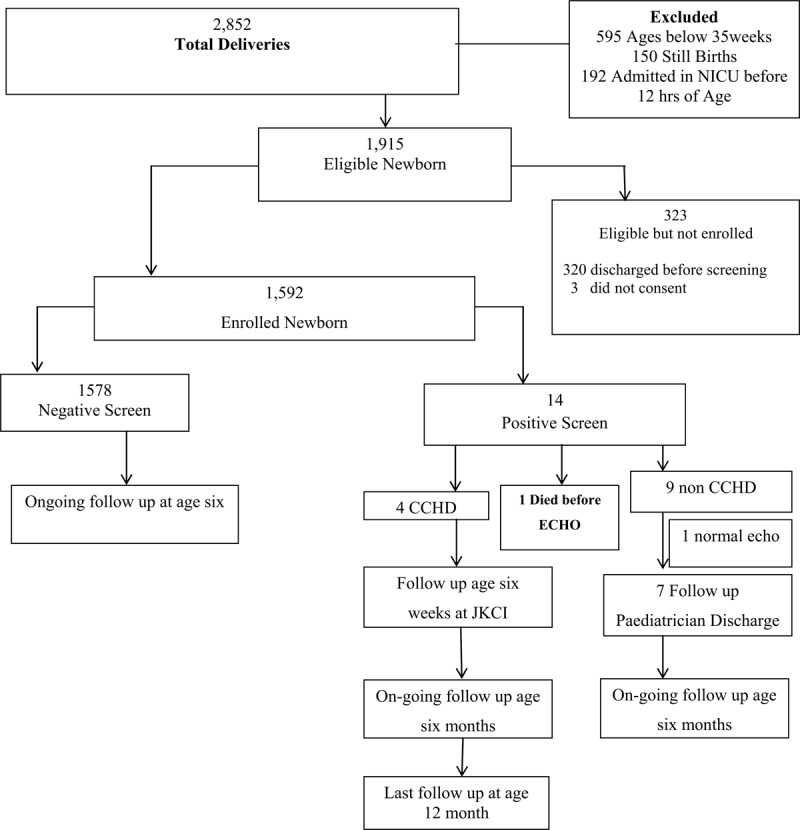
Cohort Profile.

Only MNH was actively collecting data as per the feasibility study during the initial study period before the screening program was expanded to MRH. ***Figure 2*** shows the enrolment to the ongoing follow-up of patients.

Two thousand eight hundred fifty-two infants were born at the MNH maternity ward during the study period. While 1,915 were eligible for screening, 937 did not meet the inclusion criteria. 320 (17%) were discharged before being screened and3 (0.2%) declined consent.

***Table 1*** shows characteristics of screened newborns. A total of 1,592 (83%) newborns were available for POX screening. There were 52% males and 65% were born by caesarean section. The mean weight of babies screened was 3.08 ± 1.04 kg. Approximately 8% had other associated congenital malformations.

**Table 1:** Baseline characteristics of newborns screened (N = 1592).


VARIABLE (N = 1592)	CATEGORY	NUMBER (n)	PERCENTAGE (%)

Sex	Male	834	52.4

	Female	758	47.6

Mode of delivery	Spontaneous vertex delivery	557	35.0

	Caesarean section	1032	64.8

	Breech delivery	03	0.80

Apgar score	1–4	38	2.40

	5–8	280	17.6

	9–10	1274	80.0

Obvious malformations	Yes	12	0.80

	no	1580	99.2

First Screening results	Positive screening	11	0.60

	Negative screening	1573	98.9

	Undetermined	8	0.50


There were 11 positive screens and eight undetermined test results. Three of the undetermined results were positive after retest. A total of 14 (0.9%) positive screens were referred for an Echocardiogram at JKCI (***Table 2***), which confirmed four (4/14) infants to have CCHD. The CCHD detection rate in this initial study was 2.5 per 1,000 live births at 95% confidence interval [CI], 0.9–6.7 per 1000 live births, with false positive rate of 0.6. Baby #1 had d-TGA; baby #2 neonatal co-arctation of the aorta, baby #3 with pulmonary atresia intact septum and baby #4 with a severe form of ectopic cordis double outlet right ventricle. The four infants with CCHD diagnosis continue with follow-up under protocol. Seven out of 10 false-positive infants had significant pulmonary conditions mainly of a combination of PDA and persistent pulmonary hypertension of the newborn.

**Table 2:** Characteristics of new-borns with positive screen, MNH.


GESTATION AGE(WEEKS)	MODE OF DELIVERY	ECHO DIAGNOSIS	SATURATION% (R-ARM/LEG)	STATUS AT AGE SIX WEEKS

36	C-Section	Persistent pulmonary hypertension of newborns	99%/83%	Patient Died day ten post-delivery with the discharge diagnosis of neonatal Sepsis

35	C-Section	Normal echocardiography	89%/93%	Alive, discharged on day 4

38	C-Section	Co-arctation of Aorta	93%/94%	Underwent Successful Neonatal coarctation repair at age 20 days

39	C-Section	Persistent pulmonary hypertension of newborns & PDA	92%/88%	Alive discharged on day 3

37	SVD	Persistent pulmonary hypertension of newborns & PDA	93%/97%	Alive discharged on day 10

40	SVD	Persistent pulmonary hypertension of newborns		Alive discharged on day 3

38	Breech	Transposition of great arteries	87%/67%	Alive, underwent successful Rashkind procedure followed by Arterial switch operation on day 10

38	SVD	Persistent pulmonary hypertension of newborns	97%/86%	Alive, discharged on day 4

38	SVD	Normal Echo-Sepsis	88%/92%	Alive, discharged on day 9

36	C-Section	Pulmonary Atresia Intact septum	90%/86%	Alive, underwent B.T. shunt on day 32

36	SVD	Ectopic Cordis with DORV	89%/91%	Died on day 12 from complications of neonatal sepsis

37	SVD	Died before Echo	89%/91%	Died before Echo

38	C-Section	Persistent pulmonary hypertension of newborns	66%/75%	Alive discharged on day 10

39	C-Section	Persistent pulmonary hypertension of newborns	80%/90%	Alive, discharged on day 9


**C-section:** Caesarean Section, **SVD:** Spontaneous Vertex Delivery, **PDA:** Patent Ductus Arteriosus, **DORV:** Double Outlet Right Ventricle, **R-arm:** Right arm.

***Table 3*** shows the feasibility of the ongoing study. Approximately 83% of eligible children were enrolled. The main reason for failure to enrol eligible children was discharges less than 12 hours after delivery. Nurses followed the protocol 80% of the time, and most of the screened children, 93% of whom needed echocardiography, received the confirmatory test.

**Table 3:** Feasibility of the ongoing study.


ITEM	NUMBER	PERCENTAGE	DESCRIPTION

Number of Providers available for screening vs Trained	25/30	83%	Other trained providers are administrators who are not always available for screening

Eligible Newborn	1,915/2,852	67%	Age below 35 weeks and, admission in NICU were the main reasons for non-inclusion

Enrolled Newborn	1,592/1,915	83%	320 new-borns were discharged before age 12 hours, another 3 did not consent

False Positive	10/14	71%	Majority (80%) had significant pulmonary conditions

Protocol Deviation	2/10	20%	Repeated pulse oximeter at the time of Echocardiogram showed normal saturation.

Missed Opportunity	1/14	7%	The Patient died before Echocardiogram could be performed


## Discussion

As infant mortality rates from communicable diseases decrease, newborn screening initiatives such as POX for CCHD is a growing public health priority in low and middle-income countries [26]. When coupled with an increased rate of hospital deliveries observed in developing countries [27], it offers an opportunity window to screen for diseases soon after birth. Furthermore, economic progress and emergence of cardiac centres create an avenue for studying and consequently improving the survival of the previously neglected CHD in the region [17,28,29]. Here we report our preliminary results on POX performance in a low-resource setting.

From the preliminary data the detection rate for CCHD is 2.5 per 1,000 live births at 95% confidence interval [CI], 0.9 to 6.7 per 1,000 live births. These findings match and complement previous study results from the USA [5,6,21]. Liske et al. found the rate of CCHD in Tennessee as 2.9 per 1,000 live births [21]. Reller et al. found the rate to be 2.2 per 1,000 live births in a large population-based study in 2008 conducted in Metropolitan Atlanta [5]. According to findings, CCHD occurs in Tanzania at a rate matching other parts of the world.

This study screened over 80% of eligible infants, indicative of high performance and acceptability. It is congruent to the conclusion by previously published data from South Africa by Van Niekerk et al. in 2016 that with adequate staff and wide newborn screening POX implementation in an African setting is possible without additional burden to cardiology services [13].

Apart from staff adequacy for a successful POX program, training, re-training and education to providers on how to perform, interpret results and respond to a neonate who fails the test is crucial [14]. In our study, over 80% of the nurses trained had to be actively and continuously participating daily in the screening of newborns. Moreover, nurses were allowed to start screening only after passing the competence test, which mandated a 95% pass mark, and training continued through daily support offered by the study team. This level of training and re-training was adequate, as evidenced by a low percentage of protocol deviations observed in the current study. Govender et al. observed in their study in KwaZulu-Natal, South Africa that, despite the training of all nurses in the nursery, only enrolled nurses were available for screening. Thus, low POX uptake and prolonged enrolment were seen in their study [14]. These observations suggest the need to continuously assess and support the workforce to realize the program benefits.

Once a positive screen is identified, challenges may remain on the availability of experts to perform an echocardiogram for confirmation before discharge, a challenge pertinent in a low-resource setting. However, we offered confirmatory echocardiography tests to 93% of eligible infants. The one patient who missed an Echocardiography in our case was born on a public holiday, where a cardiologist was not readily available.

The success observed in this study is partly explained by the accessibility of an echocardiogram machine at the nursery of the study hospital, emphasizing the need to have all the necessary equipment for a successful program [13,14]. Alternative proposals include arranged transport with a well-designed referral mechanism for timely confirmatory tests [32].

Appropriate timing of screening is a challenge in a low-resource setting, where the tendency for early discharge (<24 hours) after hospital delivery is standard [34,35]. It is well documented that the rate of false positives may increase in case of screening prior to normal newborn transition. Nevertheless, screening after 24 hours post-delivery or around discharge time if at least 12 hours have lapsed is widely acceptable [7,8,9,10]. A too flexible timing may reduce the false positive rate and elude normal neonatal transition, but could create a missed screening opportunity to normal deliveries with a tendency to discharge early from birth hospital [33]. In our study, almost all eligible newborns who missed screening were those discharged within 12 hours. This happened despite hospital guidelines stating that all deliveries have to be observed and discharged after 24 hours to monitor complications. Realistically, early discharges for uncomplicated deliveries are to be expected, which calls for the need to carefully balance discharge needs and screening protocol so as to realize the full benefits of a newborn screening program. It remains to be seen on the continuation of this study what the risks are of CCHD for early discharges.

This study’s false-positive rate of 0.6% is slightly higher to Norway’s 0.14% [23], or German’s 0.1% [16] but comparable to the UK’s 0.8% [18]. Poland’s 0.026 [16] is the lowest rate while Saudi Arabia’s 3.4% and Ethiopia’s 6% [24,25] top the list. Observed differences in false positive rates are basically due to prevalence and the timing for CCHD screening. In Ethiopia, higher altitude could be the culprit [25]. Our study’s false-positive rate could partially be explained by a high proportion of caesarean section in our cohort tending to have newborn high persistent pulmonary hypertension. The positive predictive value in our study was 30.1%, similar to the German (25.9%) and Sweden (30.7%) studies [16,22]. These findings indicate a similar POX performance rate in a low-resource setting.

Although the goal of screening was to detect CCHD, seven of the ten false-positive POX tests (71%) had persistent pulmonary hypertension on echocardiogram. Thus, discharge was withheld for 2–3 days supportive management (without ventilator support). Our preliminary data confirm POX’s well-established and extensively described secondary benefits [20]. By detecting other potentially severe newborn conditions, interventions were done before deterioration, a phenomenon particularly useful in developing nations where postnatal check-ups are limited and respiratory illnesses are major cause of deaths in the age group [30,31,35].

### Study Limitations

The TPOXS is ongoing and definitive conclusions are premature. Moreover, the study is performed in a tertiary and secondary care setting pointing at inconclusive observations whether the experience can be transmitted to the lower levels health facilities.

### Implications for practice

Preliminary results suggest CCHD with POX screening of newborns appears feasible with a high acceptance rate in a well-prepared limited resource setting. However, a definitive conclusion cannot be drawn. It is imperative that the timing for screening be chosen carefully to balance missed opportunities and false-positive rates. Indeed, as others previously suggested, the usefulness of POX may extend beyond CCHD detection and could aid in the early detection of other critical respiratory pathologies and thus the potential to protect more lives in developing countries.

## Conclusion

This paper provides the protocol of the TPOXS, whose preliminary results confirm adequate performance and acceptability of POX screening for CHHD in a well-prepared low-resource setting. It similarly highlights the well-proven secondary benefits of POX screening and a need to balance screening timing to capture all newborns while avoiding excess false-positive rates. Definitive conclusions will be based on complete enrolment.

## Data Accessibility Statement

The datasets analyzed during the current study are available from the corresponding author upon reasonable request.

## Additional File

The additional file for this article can be found as follows:

10.5334/gh.1110.s1 Supplementary file. Appendix 1 Bistros pulse oximeter device and Appendix 2 is neonatal.
