# Supplementary material for: Newborn Screening for Critical Congenital Heart Disease in a Low-Resource Setting; Research Protocol and Preliminary Results of the Tanzania Pulse Oximetry Study
Source: Glob Heart. 2022 May 26;17(1):32. doi: 10.5334/gh.1110 (PMC9139018; doi:10.5334/gh.1110)

Attachment 1: FINGERTIP PULSE OXIMETER BT -710 PEDIATRIC

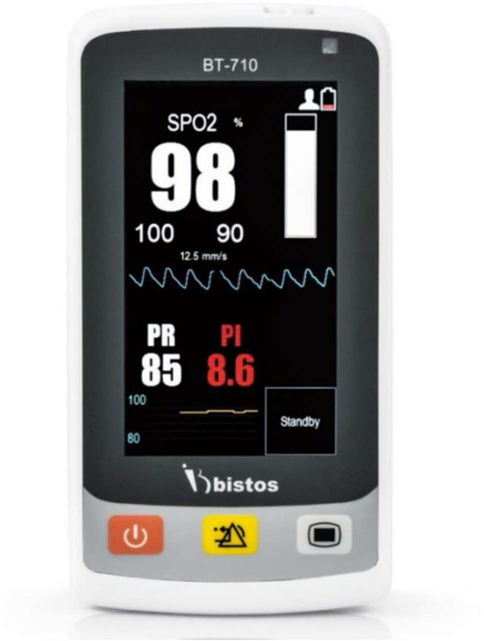

The Bistos BT-710 is a handheld pulse oximeter with a 4.3" color LCD touch screen. It provides an accurate SpO2 reading during motion and low perfusion. The re-chargeable built-in li-ion battery provides 8 hours of continuous operation and easy charging with the pulse oximeter's mini USB port. It is suitable for adult, pediatric and neonatal patients.

Attachment 2: BISTOS -710 SPO2 NEONATAL SENSOR

Re-usable, neonatal SpO2 sensor, with One meter cable.

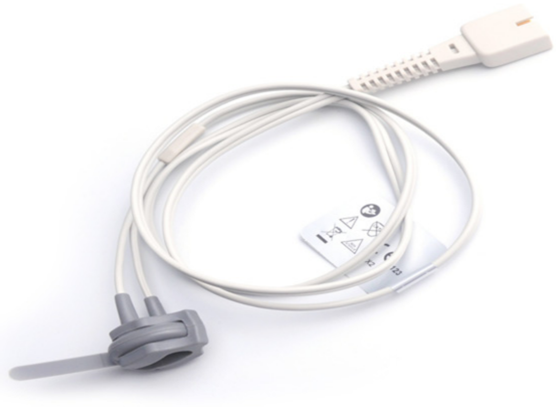

Supplement: Supplementary file. — Appendix 1 Bistros pulse oximeter device and Appendix 2 is neonatal. [file gh-17-1-1110-s1.pdf]
